# Functional Screenings Identify Regulatory Variants Associated with Breast Cancer Susceptibility

**DOI:** 10.3390/cimb43030124

**Published:** 2021-10-26

**Authors:** Naixia Ren, Yingying Li, Yulong Xiong, Panfeng Li, Yutian Ren, Qilai Huang

**Affiliations:** Shandong Provincial Key Laboratory, Animal Cell and Developmental Biology, School of Life Sciences, Shandong University, Qingdao 266237, China; naixiaren@gmail.com (N.R.); yingyingli950602@gmail.com (Y.L.); sdu.ylxiong@gmail.com (Y.X.); panfengli2021@gmail.com (P.L.); jacobren10058@gmail.com (Y.R.)

**Keywords:** regulatory SNP, breast cancer, rs4808611, *NR2F6*, rs2236007, EGR1, *PAX9*

## Abstract

Genome-wide association studies (GWAS) have identified more than 2000 single nucleotide polymorphisms (SNPs) associated with breast cancer susceptibility, most of which are located in the non-coding region. However, the causal SNPs functioning as gene regulatory elements still remain largely undisclosed. Here, we applied a Dinucleotide Parallel Reporter sequencing (DiR-seq) assay to evaluate 288 breast cancer risk SNPs in nine different breast cancer cell lines. Further multi-omics analysis with the ATAC-seq (Assay for Transposase-Accessible Chromatin using sequencing), DNase-seq (DNase I hypersensitive sites sequencing) and histone modification ChIP-seq (Chromatin Immunoprecipitation sequencing) nominated seven functional SNPs in breast cancer cells. Functional investigations show that rs4808611 affects breast cancer progression by altering the gene expression of *NR2F6*. For the other site, rs2236007, the alteration promotes the binding of the suppressive transcription factor EGR1 and results in the downregulation of *PAX9* expression. The downregulated expression of *PAX9* causes cancer malignancies and is associated with the poor prognosis of breast cancer patients. Our findings contribute to defining the functional risk SNPs and the related genes for breast cancer risk prediction.

## 1. Introduction

Breast cancer is the most commonly diagnosed malignancy and the main cause of cancer-related mortality in women [1]. As of 2020, the estimated numbers of breast cancer new cases and deaths in females are both ranked number one from a survey of the World Health Organization (WHO) [2,3]. Genome-wide association studies (GWAS) have been popular in discovering the associations between single nucleotide polymorphisms (SNPs) and diseases [4,5,6]. A mickle of GWAS has identified over 2000 breast cancer-associated SNPs, highlighting that genetic factors make a great contribution to breast cancer susceptibility. Specifically, the common genetic variations explain up to 18% of the familial relative risk for breast cancer [7]. Functional studies of the risk SNPs have resulted in successful findings of regulatory SNPs and elucidation of the thereof mechanisms in complex diseases [8,9,10,11,12,13,14,15,16,17,18,19]. Accumulating studies show that the functional risk SNP sites usually alter the chromatin binding of the transcription factors and result in the abnormal expression of the target gene [8,12,18,20,21,22,23,24,25,26]. However, the majority of these GWAS SNPs remain unclear with respect to their biological function and underlying mechanisms.

We previously developed the versatile Dinucleotide Reporter Assay system (DiR-seq) for screening regulatory SNPs in a parallel manner [27] and successfully identified multiple causal SNPs [18,19]. On the other hand, gene regulatory elements are usually marked as DNA hypersensitive sites and the histone modifications such as H3K4me3 and H3K27ac [8,28,29]. Hence, the application of the multi-omics data of the DNase-seq and histone modification ChIP-seq in combination with gene reporter analysis will help identify the functional SNP sites more accurately.

*NR2F6* was widely studied as the nuclear receptor subfamily in the field of cancer immunology, especially T-cell responses [30,31]. Moreover, *NR2F6* has been reported to be associated with cell growth and differentiation in leukemia [32] and the progression of colorectal cancer [33]. Although *NR2F6* was identified to be the hub genes involved in the pathogenesis and progression of breast cancer [34,35], the regulatory mechanisms in breast cancer are unclear. EGR1 is a C2H2 zinc finger protein of the EGR family and contains a highly conserved DNA binding domain that binds to GC-rich motifs. Depending on the binding site and co-factors, it binds to the gene promoters to either activate or suppress gene transcription [36,37]. It has been reported that PAX9 protein interacts with the nuclear protein PLU-1 and plays a vital role in the development of breast cancer malignancies [38]. In addition, progressive loss of *PAX9* expression correlates with increased malignancy in esophageal cancers [39].

Here, we performed the DiR-seq analysis to screen breast cancer risk-related SNPs in nine different breast cancer cell lines. We disclosed the strong cell-specific profiles with the gene regulatory activity of the 288 SNPs. In addition, mapping these risk SNPs to ATAC-seq, DNase-seq and H3K4me3/H3K27ac cistromes in MCF7 and T-47D cells allow us to nominate the seven most functional SNP sites. Further functional studies focusing on the rs4808611 and rs2236007 sites revealed their roles in cancer risk and shed light on the underlying molecular mechanism. We found that the C allele of rs4808611 may affect the risk progression of breast cancer by promoting the expression of *NR2F6* (HGNC:7977). Meanwhile, the rs2236007 site can decrease the expression level of a breast cancer-related gene, *PAX9* (HGNC:8623), via altering the binding of the repressive transcription factor, EGR1. The downregulated expression of the *PAX9* gene will finally result in a poor prognosis of breast cancer patients.

## 2. Materials and Methods

### 2.1. DiR-seq Plasmid Library Construction for Breast Cancer Risk Variants

We enlisted 285 breast cancer-associated risk tag SNPs (*p*-value < 10^−5^) (Table S1) from the publicly available GWAS catalog in 2016 (https://www.ebi.ac.uk/gwas/, accessed on 1 August 2016) as well as three breast cancer susceptibility SNPs (Table S2) extracted from a review paper [40]. We obtained the 55bp SNP-centered DNA sequences from the human genome (GRCh38/hg38) on the UCSC genome browser. The corresponding oligos (Table S3) were ordered, annealed, and then inserted into the DiR-Promoter vector between BglII (FD0083, Thermo Scientific, Waltham, MA, USA) and SmaI (FD0664, Thermo Scientific, Waltham, MA, USA) sites with T4 DNA Ligase (EL0011, Thermo Scientific, Waltham, MA, USA) as described previously [27]. Each construct was confirmed by Sanger sequencing. Finally, we obtained 576 successful constructs for 288 SNPs (Table S4). The 576 reporter constructs for 288 SNPs were mixed with the DiR blank vector and used for DiR reporter assay in breast cancer cells.

### 2.2. Cell Culture

Breast cancer cell lines (MCF7, ZR-75-1, and T-47D) were grown in the RPMI-1640 (Gibco, New York, NY, USA) medium with 10% FBS (Gibco, New York, NY, USA), 12.5 mM HEPES (Sigma, St. Louis, MO, USA) and 1% antibiotics (Penicillin-Streptomycin, Sigma, St. Louis, MO, USA). BT-549 was grown in the PRMI-1640 (Gibco, New York, NY, USA) medium with 0.023 U/mL insulin, 10% FBS (Gibco, New York, NY, USA), 12.5 mM HEPES (Sigma, St. Louis, MO, USA), and 1% Penicillin-Streptomycin (Sigma, St. Louis, MO, USA). SK-BR-3 was grown in McCoy’s 5a medium modified RPMI-1640 (Gibco, New York, NY, USA) medium with 10% FBS (Gibco, New York, NY, USA), 12.5 mM HEPES (Sigma, St. Louis, MO, USA), and 1% Penicillin-Streptomycin (Sigma, St. Louis, MO, USA). MDA-MB-468 was grown in DMEM (Gibco, New York, NY, USA) medium with 10% FBS (Gibco, New York, NY, USA) and 1% Penicillin-Streptomycin (Sigma, St. Louis, MO, USA). MDA-MB-453 and BT-474 were grown in DMEM/F12 (Gibco, New York, NY, USA) medium 10% FBS (Gibco, New York, NY, USA) and 1% Penicillin-Streptomycin (Sigma, St. Louis, MO, USA). BT-20 was grown in EMEM (Gibco, New York, NY, USA) medium 10% FBS (Gibco, New York, NY, USA) and 1% Penicillin-Streptomycin (Sigma, St. Louis, MO, USA). All the above cell lines were purchased from the American Type Culture Collection (ATCC) and tested negative for mycoplasma with the Myco-Blue Mycoplasma Detector (D101-01, Vazyme, Nanjing, China). All cell lines used in this study were cultured following the instructions outlined in ATCC.

### 2.3. Cell Transfection

The DiR-seq plasmid library was extracted using the plasmid miniprep plus purification kit (GeneMark, Taiwan, China) and subjected to cell transfection with Lipofectamine 2000 Reagent (11668-019, Invitrogen, Carlsbad, CA, USA) following the manufacturer’s instructions. Each transfection experiment was repeated three times independently. Cells were seeded in a 12-well plate and transfected on the next day when the cell reached 70–90% confluence. The 1 μg DNA and 3 μL transfection reagent that had been diluted separately in Opti-MEM (Gibco, New York, NY, USA) were combined and added to cells by drops after 10–15 min incubation. The cells were harvested at 24–48 h post-transfection and subjected to RNA isolation.

### 2.4. RNA Isolation and Reverse Transcription

The cells were washed twice and harvested in 1 × PBS, and total RNA was extracted using RNeasy Plus Mini Kit (74136, QIAGEN, Dusseldorf, Germany). We treated 2 μg RNA samples with Rapidout DNA Removal Kit (Thermo Scientific, Waltham, MA, USA) for 60 min at 37 °C to remove trace genomic DNA contamination according to the kit protocol. The purified RNA was used for reverse transcription with High-Capacity cDNA Reverse Transcription Kits (4374967, Applied Biosystems, MA, USA). Briefly, 1.5 μg RNA sample was applied in a 20 μL reaction and incubated at 25 °C for 10 min, followed by 120 min at 37 °C. Then, the reverse transcriptase was inactivated by heating to 85 °C for 5 min. Finally, the cDNA product was stored at –20 °C or –80 °C for qPCR analysis and next-generation sequencing (NGS) library preparation. For the DiR reporter assay, the sequence-specific primer BarP6 (Table S7) was used in the reverse transcription, while the random primer included in the High-Capacity cDNA Reverse Transcription Kits was used for reverse transcription and other applications.

### 2.5. DiR-seq NGS Library Construction and Sequencing

The constructions for NGS libraries were performed as previously described [19]. We used 2× Phusion Hot Start II High-Fidelity PCR Master Mix (Thermo Scientific, Waltham, MA, USA) to perform two rounds of PCR. The first round of PCR introduces the binding sites of Illumina sequencing primers at both ends of the product. In order to comply with the 150 bp paired-end sequencing strategy of the Illumina HiSeq X-TEN platform, the 450 bp barcode sequence was divided into a 271 bp first half amplicon and a 270 bp second half amplicon. The purpose of the second round of PCR was to introduce adaptors for cluster generation and the index sequences. The template used in the second round of PCR was from every twelve sets of the first-round PCR products. We used 24 sets of primers in the first round of PCR and 12 sequencing indexes in the second round of PCR to obtain up to 288 sub-libraries in one NGS library. The plasmid pool was constructed as described above for the input control. We used the 150 bp paired-end sequencing on the Illumina HiSeq X-TEN platform for the libraries. Primers used for the construction are shown in Table S5.

### 2.6. DiR-seq Data Analyses

We processed the DiR-seq data as described previously [18]. Briefly, we used the software ‘FastP’ [41] and ‘Panda-seq’ [42] to clean and assemble the paired reads and used the R package ‘ShortRead’ [43] to sort out the sub-libraries. We counted the barcode reads for each sub-library using the R package ‘ShortRead’. In order to eliminate the influence of the sequencing depth in different sub-libraries, we normalized the barcode counts to one million for each library. Then, the expression level of the reporter gene was defined by using the normalized reads from RNA divided by the normalized reads from the DNA template. We first selected the SNP sites that drove reporter expression differently with both alleles (*p* < 0.05). Then, we further defined regulatory SNP sites with the criteria that the reporter expression level of at least one allele was lower than 0.8 or higher than 1.2. All the nominated SNPs in different breast cancer cells were listed in Table S6.

### 2.7. Quantitative PCR

The AceQ qPCR SYBR Green Master Mix (Q111-03, Vazyme, Nanjing, China) was used in qPCR assay and cycled on thermocyclers Rotor-Gene Q (Qiagen, Dusseldorf, Germany) or LightCycler 96 thermal cycler Instrument (Roche Applied Science, Indianapolis, IN, USA). All the primer pairs used in the qPCR assay had confirmed to have a perfect specificity and amplification efficiency. All the qPCR assays were performed in three technical replications. For analysis of mRNA expression, we normalized the expression data against the endogenous *ACTB* (*β-actin*) control. For ChIP qPCR and FAIRE qPCR assays, the relative enrichment of the interested DNA region was calculated over the input control and then normalized to the control region. Moreover, for the AS-qPCR assays, primers were designed for allele-specific amplification by positioning the allele-specific nucleotide at the 3′ end. The DiR-seq qPCR primers were listed in Table S7, and all the other qPCR primers were listed in Table S8.

### 2.8. Formaldehyde-Assisted Isolation of Regulatory Elements (FAIRE)

The FAIRE assay was performed as previously described with slight modifications [44]. Briefly, breast cancer cells were fixed with 1% formaldehyde (F8775, Sigma-Aldrich, St. Louis, MO, USA) for 10 min at room temperature and then quenched with a final concentration of 125 mM glycine (0167, Amresco Radnor, PA, USA). After twice washing with cold PBS, the fixed cells were collected and stored at –80 °C or were used immediately. The cell pellet was resuspended in hypotonic lysis buffer (20 mM Tris-HCl, pH 8.0, with 10 mM KCl, 10% glycerol, and 2 mM DTT supplied with complete EDTA-free Protease Inhibitor Cocktail) and incubated at 4 °C for 30 min with rotation. The cell nuclei were then collected by centrifugation for 5 min at 5000× *g* at 4 °C and washed with 1 mL cold PBS. The pellet was resuspended in 2% SDS lysis buffer (50 mM Tris-HCl, pH 8.1, with 2% SDS and 10 mM EDTA supplied with complete EDTA-free Protease Inhibitor Cocktail) and incubated at 4 °C for 30–60 min. The chromatin was sonicated to an average size of about 200 bp with a Bioruptor (Bioruptor pico, Rue Bois Saint, Belgium). The chromatin lysate was cleared by centrifugation at 13,000× *g* for 5 min at 4 °C, and the given amount of lysate containing 0.5 µg chromatin DNA was then subjected to double phenol/chloroform/isoamyl alcohol extraction followed by one chloroform/isoamyl alcohol extraction. The aqueous (top) layer was transferred to fresh 1.5 mL tubes, and 20 µg glycogen (Thermo Scientific, Waltham, MA, USA) was added to each tube, followed by incubation at −80 °C for 30 min or longer. The DNA was centrifuged and resuspended in 10 mM Tris-HCl (pH 7.4). FAIRE DNA and Input DNA control were treated with RNase A (Thermo Scientific, Waltham, MA, USA) at a final concentration of 0.2 mg/mL at 37 °C for 30 min. Then, FAIRE DNA and Input DNA were subjected to reverse cross-linking overnight at 65 °C with proteinase K (Thermo Scientific, Waltham, MA, USA) and purified using 1 × VAHTS DNA Clean Beads and were ready for qPCR analysis or PCR amplification for Sanger sequencing. Importantly, all the tubes in this experiment were low adhesion, and all primers used for the FAIRE assay are listed in Table S8.

### 2.9. Chromatin Immunoprecipitation (ChIP)

ChIP assay was performed as previously described with slight modifications [8]. Briefly, sheared chromatin was obtained as described above in the FAIRE analysis. The chromatin lysate was applied to immunoprecipitation with antibodies against H3K4me3 (ab8580, Abcam, Cambridge, UK), H3K27ac (ab4729-100, Abcam, Cambridge, UK), EGR1 (Early growth response 1) (4153S, CST, Danvers, MA, USA) or normal rabbit IgG (2729, CST, Danvers, MA, USA). The antibodies were pre-coated with Magna ChIP Protein A + G Magnetic Beads (16-663, EMD Millipore, St. Louis, MO, USA) in the blocking buffer that contains 0.5% BSA in IP buffer (20 mM Tris-HCl, pH 8.0, with 2 mM EDTA, 150 mM NaCl, 1% Triton X-100 supplied with complete EDTA-free Protease Inhibitor Cocktail). Antibody-beads-DNA complexes were washed twice in turn with wash buffer I (20 mM Tris-HCl, pH 8.0, with 2 mM EDTA, 0.1% SDS, 1% Triton X-100 and 150 mM NaCl), wash buffer II (20 mM Tris-HCl, pH 8.0, with 2 mM EDTA, 0.1% SDS, 1% Triton X-100 and 500 mM NaCl), wash buffer III (10 mM Tris-HCl, pH 8.0, with 1 mM EDTA, 250 mM lithium chloride, 1% deoxycholate and 1% NP-40) and buffer IV (10 mM Tris-HCl, pH 8.0 and 1 mM EDTA). DNA-protein complexes were extracted with extraction buffer (10 mM Tris-HCl, pH 8.0, with 1 mM EDTA and 1% SDS) at 65 °C and incubated with RNase A (Thermo Scientific, Waltham, MA, USA) at a final concentration of 0.2 mg/mL for 30 min at 37 °C. Then, the DNA-protein complexes were reverse cross-linked overnight at 65 °C with proteinase K (Thermo Scientific, Waltham, MA, USA). The DNA was purified using 1 × VAHTS DNA Clean Beads for qPCR analysis or Sanger sequencing analysis of PCR. All primers used for the ChIP assay are shown in Table S8.

### 2.10. Genome Editing through CRISPR/Cas9

Genome editing experiments were performed according to the previous protocol [45]. The single guide RNA (sgRNA) targeting the aimed site, or a non-mammalian control sequence, was designed based on the NGG protospacer adjacent motif (PAM) of S. pyogenes Cas9. The annealed oligos were inserted into the BbsI-linearized pSpCas9(BB)-2A-Puro (PX459) V2.0 (62988, Addgene) vector. All the gRNA oligos sequences are listed in Table S9. The resulting plasmids were transfected into the breast cancer cells, including MCF7, MDA-MB-453, and MDA-MB468, in a 12-well plate at a 70% confluence. Transfection was performed using a Lipofectamine 2000 Transfection Reagent (11668-019, Invitrogen, Carlsbad, CA, USA) according to the instructions of the manufacturer. The medium was replaced with the fresh medium containing 2 μg/mL puromycin 24–48 h post-transfection. When the non-transfected cells died, the surviving cells were collected for editing efficiency evaluation by getPCR analysis or gene expression evaluation by RT-qPCR.

### 2.11. Genome Editing Efficiency Determination and Single-Cell Clone Screening

We assessed genome editing efficiency using the getPCR method as previously described [46]. Briefly, the tested primers for getPCR were designed with four watching nucleotides over the cutting site. The 15 μL reaction system of AceQ qPCR SYBR Green Master Mix (Vazyme, Nanjing, China) was cycled with a 95 °C for 5 min, followed by 45 cycles of 95 °C for 15 s, 69 °C for 15 s and 72 °C for 15 s on a Roche LightCycler96. While screening the single-cell clones, the watching primers with 3’ end located on the cutting site were used for the qPCR test. Notice that we designed a control amplification about 200 bp away from the cutting site for normalization in calculating the percentage of wildtype DNA in the edited genomic DNA. The primers used in getPCR experiments are listed in Table S8.

### 2.12. EGR1 Gene Knockdown and Overexpression

For gene knocking down of *EGR1*, we used the pLKO.1-puro vector to express shRNA, the sequence of which comes from the validated shRNA clones in MISSION^®®^ shRNA Library (Sigma-Aldrich, St. Louis, MO, USA). The oligos bearing shRNA sequence (Table S9) were annealed and inserted into a pLKO.1-puro vector that has been cleaved with EcoRI (FD0274, Thermo Scientific, Waltham, MA, USA) and BshTI (FD1464, Thermo Scientific, Waltham, MA, USA). For the overexpression of EGR1, the CDS region was amplified from MCF7 cDNA using primers listed in Table S9 and cloned into pcDNA™3.1 (V810-20, Invitrogen, Carlsbad, CA, USA) between KpnI and XbaI. The shRNA plasmid and overexpression plasmid were transfected into MCF7 cells at 70–90% confluence in a 12-well plate using the Lipofectamine 2000 Reagent (11668-019, Invitrogen, Carlsbad, CA, USA) and following the manufacturer’s instructions. The medium was changed with the fresh medium 8 h post-transfection. The transfected cells were washed twice with 1×PBS 48 h post-transfection and collected for the gene expression test. All the primers for the gene expression test are listed in Table S8.

### 2.13. Cell Viability and Proliferation Assays

Breast cancer cells MCF7 edited with the CRISPR/Cas9 method were counted and seeded into 96-well cell culture plates at 5 × 10^3^ per well. Following the manufacturer’s instructions, cell viability and proliferation were measured with PrestoBlue™ Cell Viability (A13261, Invitrogen, Carlsbad, CA, USA) every 24 h. Fluorescence was read 2 h post adding the reagent, using an excitation wavelength of 560 nm and an emission of 600 nm on a Microplate spectrometer (PE) (Manchester, UK). The results were obtained from three biological replicates. 

### 2.14. CRISPRi and CRISPRa Analysis

To test the regulatory effect of the SNP, we co-transfected the sgRNA plasmid targeting both A and G alleles of rs2236007 with the modified vector dCas9-KRAB or dCas9-4xVP64 [47] in MCF7 cells. The transfections were performed at 70% confluence in the 12-well plate using Lipofectamine 2000 Transfection Reagent (11668-019, Invitrogen, Carlsbad, CA, USA). Forty-eight hours after transfection, the total RNA was isolated for qPCR to test the expression of the target gene. We performed each transfection in triplicates. 

### 2.15. Statistical Analysis

We adopted a two-tailed Student’s *t*-test with the Mean ± SD for all the statistical analyses except for the survival analysis. Details for the statistical method, the number of data points, and the number of replicates are indicated in each figure legend.

For hierarchical cluster analysis of gene expression profiles, we downloaded the gene expression data of nine breast cancer cells from Richard M. Neve’s work [48], and the data were processed as described using Cluster 3.0 [49]. Agglomerative clustering was applied to genes and cell lines using uncentered Pearson’s correlations. The resulted clusters were visualized using Java TreeView [50]. Hierarchical cluster analysis for our DiR-seq allele report activity was conducted in the same method.

For the multi-omics analysis, two types of data were downloaded from ENCODE (Encyclopedia of DNA Elements) [51] or GEO (Gene Expression Omnibus) [52]. The BigWig files were used for the chord plot analysis, and the R package “Goplot” was used to draw the chord plot graph. The Bed files were used for locus visualization using IGV [53].

For survival analysis in breast cancer clinical patients, we downloaded the integrated TCGA (The Cancer Genome Atlas) Pan-Cancer Clinical Data from Liu’s work [54] and merged the breast cancer clinical data to the gene expression matrix of breast cancer tissues. We used R package “survival” and “survminer” to draw a Kaplan–Meier survival plot for survival analysis. The patients were subgrouped based on the optimal cut point for the most significant relationship with survival. Meanwhile, we evaluated the hazard ratio (HR) and log-rank test using the Cox proportional hazards model to assess the statistical significance between the two groups of breast cancer clinical patients. For the Kaplan–Meier plotter online analysis [55], we split patients according to gene expression level by choosing the “auto select best cutoff”, and the Kaplan-Meier survival plots were generated, and the hazard ratios with 95% confidence intervals and log-rank *p*-value were calculated [56].

R-4.0.2 was used for the R packages. Based on all the above analyses, differences were considered to be significant when the *p*-value was <0.05.

## 3. Results

### 3.1. DiR-seq Identified Breast Cancer Risk-Associated Functional Variants

**Figure 1:**
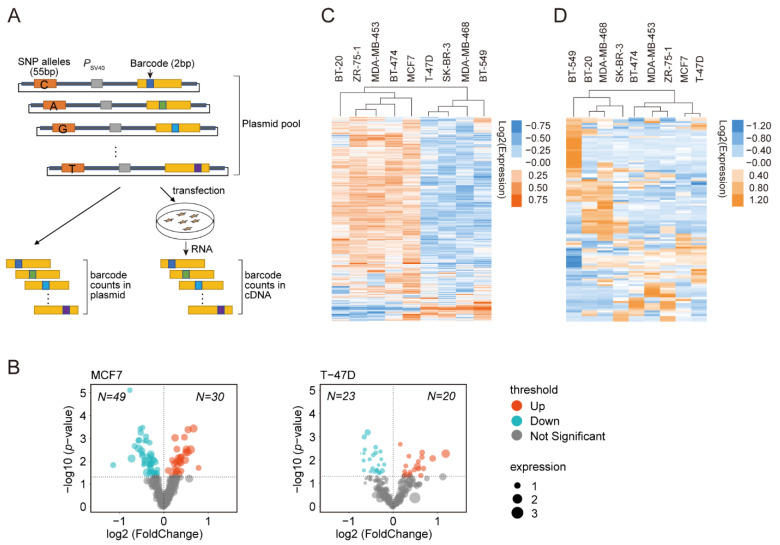
DiR-seq analysis in breast cancer cells. (**A**) DiR-seq workflow. The 55 bp genomic DNA regions carrying each allele at the center were inserted upstream of the SV40 promoter in the DiR-Promoter vectors. The resulting DiR-seq plasmid libraries were then transfected into breast cancer cells, and NGS libraries were then prepared from cDNA and template DNA. The expression level of each reporter tag was determined by counting the corresponding NGS read number in cDNA and then calibrated with the value from template DNA. (**B**) Volcano plots of DiR-seq results for the MCF7 and T-47D cells. *p* values came from a two-tailed Student’s *t*-test of effect sizes of two alleles. Dashed horizontal lines indicate the *p*-value < 0.05 cutoff. The regulatory SNPs exhibiting increased activity with the risk allele are shown in orange; those with decreased activity are shown in blue. Gray dots represent SNPs showing no significant difference between alleles. (**C**) Hierarchical cluster analysis of DiR-seq report activity for nine breast cancer cell lines. Each row represents one SNP allele, and each column represents one cell line. As shown in the color bar, white represents no change, orange represents upregulation and blue represents downregulation of the reported activity. The expression levels of each allele are shown with row normalization using *Z*-score. (**D**) Hierarchical cluster analysis of genome-wide gene expression profiles of nine breast cancer cell lines. Genes were restricted to those showing significant variance across nine cell lines, resulting in the clustering of 109 genes. Each row represents a gene, and each column represents a cell line. As shown in the color bar, white represents no change, orange represents upregulation and blue represents downregulation of gene expression. The expression levels of each gene are shown with row normalization using *Z*-score.

We performed DiR-seq analysis as described previously (Figure 1A) [18,19,27] to identify breast cancer risk-associated variants displaying transcriptional function. A total of 288 SNPs (Table S4) that have been reported for associations with breast cancer susceptibility were enlisted in the DiR-seq analysis. The DiR-seq reporter library was constructed by inserting the 55 bp genomic sequence encompassing the risk or normal allele to the upstream of the SV40 promoter in the DiR-Promoter vector (Figure 1A), with the blank DiR-Promoter construct included as a control. The 55 bp DNA fragments will generally be enough to assess the gene regulatory effect of most SNPs [57,58], even though we may miss the SNPs involving large genomic DNA regions or interactions with other factors bound on distal regulatory elements. 

The DiR-seq analysis was performed in nine breast cancer cell lines, including MCF7, ZR-75-1, MDA-MB-453, BT-20, BT-474, MDA-MB-468, T-47D, SK-BR-3, and BT-549. Correlation analysis of the tag counts in three biological replicates showed extremely high consistency in all nine cell lines (Figure S1A–I). Meanwhile, the scatterplots of tag counts between RNA samples and template DNA disclosed the gene regulatory activity for a serial of SNP sites (Figure S1J–R). Then, we used a volcano plot to display the functional variants that significantly affected gene expression (*p*-value < 0.05, Fold change = risk allele expression level/normal allele expression level) (Figure 1B, Figure S2). Specifically, in the MCF7 breast cancer cell line, 49 SNPs exhibited decreased transcriptional activity for the risk alleles (risk/normal < 1, *p* < 0.05), and 30 SNPs showed increased transcriptional activity to the contrary (risk/normal > 1, *p* < 0.05). In the T-47D cells, 43 regulatory SNPs were identified (23 decreased and 20 increased) (Figure 1B). Similarly, multiple regulatory SNPs were also nominated in the other seven breast cancer cell lines (Figure S2). In order to further choose the most hopeful regulatory SNP sites, we applied extra criteria that the reporter expression levels were less than 0.8 or higher than 1.2 relative to the blank DiR-Promoter vector. In this manner, we picked out 56 regulatory SNPs in the MCF7 cell line and 72 in the ZR-75-1 cell line, 112 in the MDA-MB-453 cell line, 63 in the BT-20 cell line, 99 in BT-474 cell line, 21 in the T-47D cell line, 36 in SK-BR-3 cell line, 36 in MDA-MB-468 cell line and 19 in BT-549 cell line, as listed in Table S6. 

Hierarchical clustering of the allele-specific reporter activity in DiR-seq analysis showed substantial variation across the nine breast cell lines. Interestingly, the clustering revealed two major branches, with one including BT-20, ZR-75-1, MDA-MB-453, BT-474, and MCF7 and the other including T-47D SK-BR-3, MDA-MB-468, and BT-549 (Figure 1C). We then obtained the genome-wide gene expression profiles of the nine breast cancer cells from Richard M. Neve’s work [48] and performed hierarchical clustering accordingly. Notably, the nine cell lines were clustered similarly in two major branches, with one including BT-474, MDA-MB-453, ZR-75-1, MCF7, and T-47D and the other including BT-549, BT-20, MDA-MB-468, and SK-BR-3 (Figure 1D). The two clustering patterns were highly consistent with the exception of the BT-20 and T-47D cells. It strongly indicates that the reporter activities from the DiR-seq analysis should have reflected the real activities of the given SNPs.

### 3.2. Multi-Omics Analysis Further Nominated Seven Most Plausible Variants

**Figure 2:**
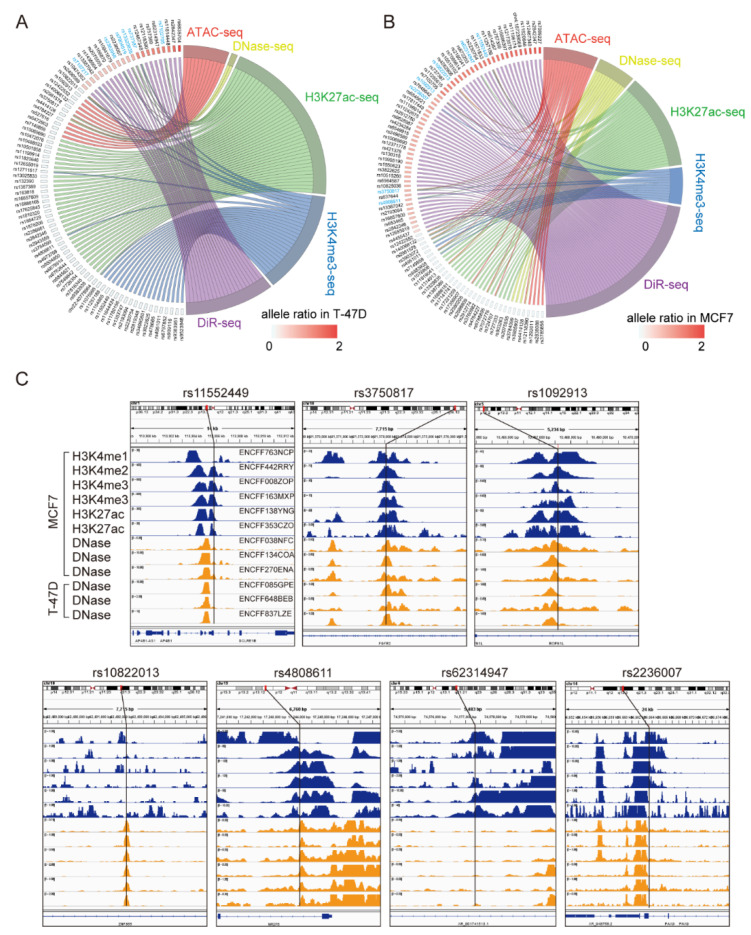
Multi-omics analysis nominated the seven most plausible functional variants. (**A**) Chord plot depicts the putative functional SNPs in DiR-seq analysis of T-47D cells and relevant omics signatures, including ATAC-seq (GSE120162), Dnase-seq (ENCFF106BSS), H3K27ac-seq (GSM2862200) and H3K4me3 (GSM2862200) obtained from ENCODE database or GEO database. Six functional SNPs nominated in T-47D cells were shown in blue. (**B**) Chord plot depicts the putative functional SNPs in DiR-seq analysis of MCF7 cells and relevant omics signatures, including ATAC-seq (ENCFF817LPT), Dnase-seq (ENCFF414CWD), H3K27ac-seq (ENCFF277GNV) and H3K4me3 (ENCFF237HYI) obtained from ENCODE database. Seven SNPs picked out in MCF7 cells were shown in blue. (**C**) Enrichment visualization for chromatin active marker at seven functional SNPs in MCF7 or T-47D. ChIP-seq data for histone modification (H3K4me1; H3K4me2; H3K4me3; H3K27ac indicating enhancer marks) in MCF7 are shown in blue. DNase-seq data in MCF7 and T-47D are shown in orange. All these data were obtained from ENCODE database, and the accession number of each experiment is shown in the first panel of rs11552449, including ENCFF837LZE, ENCFF648BEB, ENCFF085GPE, ENCFF270ENA, ENCFF134COA, ENCFF038NFC, ENCFF353CZO, ENCFF138YNG, ENCFF163MXP, ENCFF008ZOP, ENCFF442RRY and ENCFF763NCP. Genomic information is shown at the upper part, and gene information is shown at the lower part. The position of SNP in signal tracks is shown in a vertical line.

**Figure 3:**
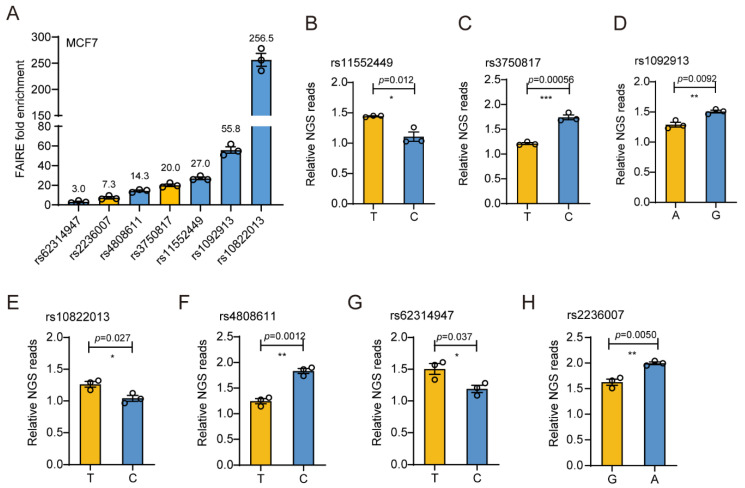
Chromatin openness analysis and gene regulatory activity analysis of seven functional SNPs in MCF7 cells. (**A**) FAIRE enrichment analysis of seven functional SNPs by FAIRE-qPCR in MCF7. Heterozygous SNPs are highlighted in orange. Mean ± SD of three technical replicates. (**B**–**H**) The reporter activity of both alleles of rs11552449 (**B**), rs3750817 (**C**), rs1092913 (**D**), rs10822013 (**E**), rs4808611 (**F**), rs62314947 (**G**) and rs2236007 (**H**) in DiR-seq analysis in MCF7. Mean ± SD of three biological replicates. * *p* < 0.05, ** *p* < 0.01, *** *p* < 0.001, two-tailed Student’s *t*-test.

In order to further prioritize regulatory functional variants that contribute to breast cancer risk in breast cancer cells, we turned to the multi-omics analysis by using whole-genome data that depicted genome-wide chromatin activity from ENCODE [51] or GEO [52] in T-47D and MCF7 cells. Among them, ATAC-seq and DNase-seq data indicate open chromatin or accessible chromatin status, and the ChIP-seq data for H3K27ac and H3K4me3 usually indicate enhancer or promoter elements. When we mapped the functional SNPs nominated by DiR-seq analysis to the multi-omics data, six sites were picked out in the T-47D cell line (Figure 2A and Figure S3A, and Table S10) and seven in the MCF7 cell line (Figure 2B and Figure S3B, and Table S11) for having active chromatin support. Moreover, further visualization in the omics disclosed the obvious enhancer signatures for the seven best SNPs, including rs11552449, rs3750817, rs1092913, rs10822013, rs4808611, rs62314947 and rs2236007 (Figure 2C). Furthermore, we evaluated the selected regulatory SNPs by using the FAIRE qPCR method and found that the seven best SNPs all have significant enrichment in MCF7 FAIRE DNA (Figure 3A). The seven SNPs have manifested significant allele-specific transcriptional functions in DiR-seq analysis (Figure 3B–H).

### 3.3. Clinical Impacts of the Related Genes of the Seven Variants

**Figure 4:**
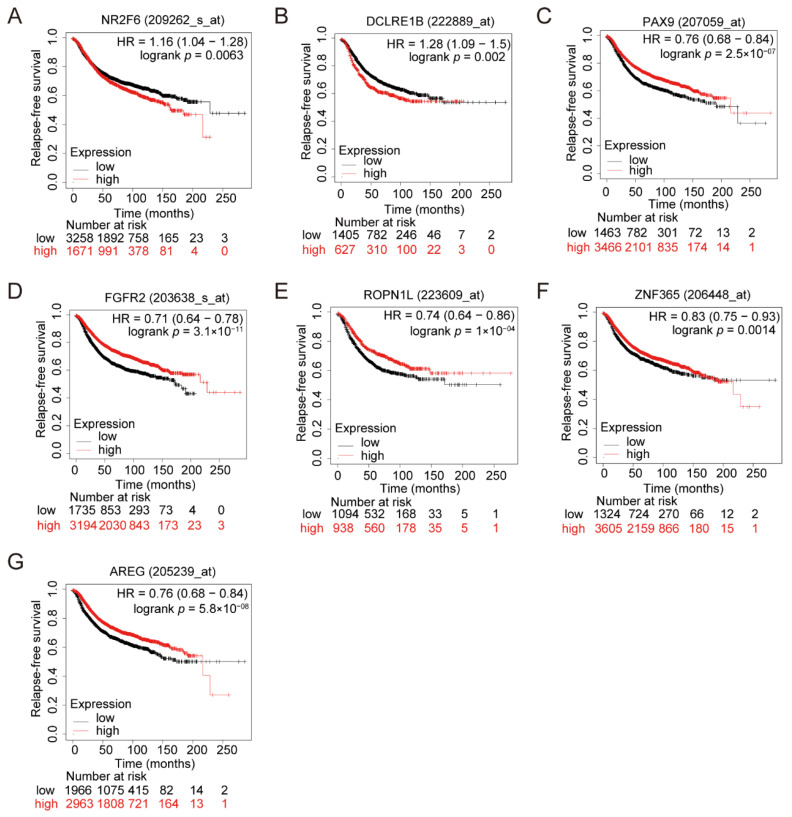
Effect of the seven risk SNP-related genes on patient prognosis. (**A**–**G**) Kaplan–Meier Survival analysis showing the relapse-free survival of breast cancer patients from GEO and EGA (European Genome-phenome Archive), stratified using the auto-select best cutoff for the expression level of *NR2F6* (**A**), *DCLRE1B* (**B**), *PAX9* (**C**), *FGFR2* (**D**), *ROPN1L* (**E**), *ZNF365* (**F**) and *AREG* (**G**). The hazard ratio for cancer relapse was provided with 95% confidential intervals, and *p* values were calculated with the log-rank test.

The rs4808611 site in 19p13.11 loci, one intron variant in the *NR2F6* gene, has been associated with breast cancer risk [40]. The Kaplan–Meier analysis showed that the higher expression of the *NR2F6* gene was associated with poor relapse-free survival for breast cancer patients (Figure 4A). The rs11552449, a missense variant in the first exon of gene *DCLRE1B* (HGNC:17641) in the 1p13.2 loci, has been reported for association with breast cancer risk [59,60,61]. Its alternative allele could significantly change the promoter activities of target gene *DCLRE1B* compared to reference alleles [62]. Our Kaplan–Meier analysis showed that the higher expression of the *DCLRE1B* gene was associated with poorer relapse-free survival for breast cancer patients (Figure 4B). When it turns to the site rs2236007, located in the 14q13.3 loci, the lower expression of the related gene *PAX9* [59,60,61,62,63] was associated with poor relapse-free survival for breast cancer patients (Figure 4C). Similarly, the rs3750817-related gene *FGFR2* (HGNC:3689) [64,65,66,67,68,69,70,71], the rs1092913-related gene *ROPN1L* (HGNC:24060), the rs10822013-related gene *ZNF365* (HGNC:18194) and the rs62314947-related gene *AREG* (HGNC:651) also exhibited association with poor relapse-free survival in breast cancer patients with lower gene expression (Figure 4D–G). 

### 3.4. The Gene Regulatory Activity of rs4808611

**Figure 5:**
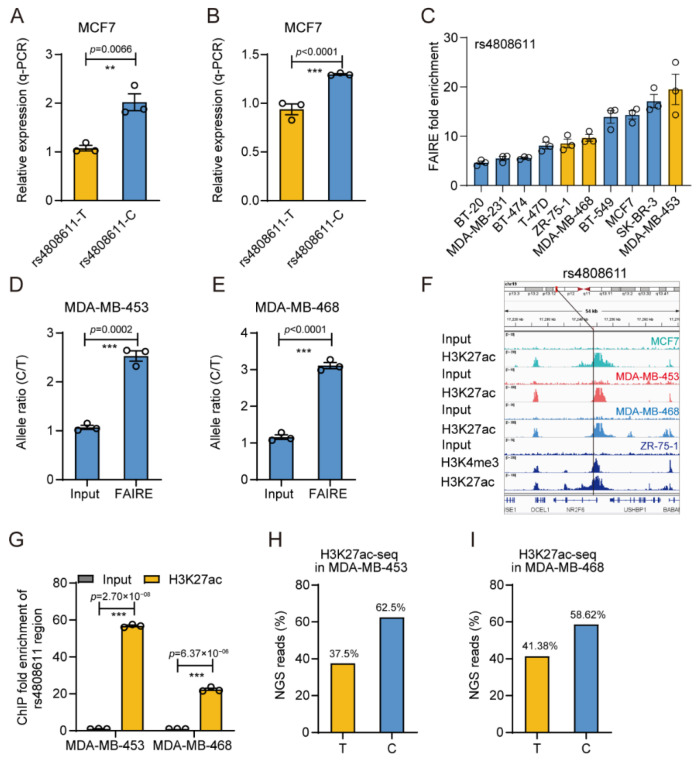
The gene regulatory activity analysis of rs4808611. (**A**) Reporter gene expression level of the rs4808611-centered 55bp region in the DiR-qPCR assay in MCF7 cells. Mean ± SD of three independent experiments. ** *p* < 0.01, two-tailed Student’s *t*-test. (**B**) Reporter gene expression level of the rs4808611-centered 733bp region in the DiR-qPCR assay in MCF7 cells. Mean ± SD of three independent experiments. *** *p* < 0.001, two-tailed Student’s *t*-test. (**C**) FAIRE-qPCR analysis of the regulatory SNP rs4808611 in ten breast cancer cells, including BT-20, MDA-MB-231, BT-474, T-47D, ZR-75-1, MDA-MB-468, BT-549, MCF7, SK-BR-3 and MDA-MB-453. The three cell lines heterozygous for rs4808611 are highlighted in orange. Mean ± SD of three independent experiments. (**D**) Allele-specific enrichment of rs4808611 region in FAIRE DNA determined by AS-qPCR in MDA-MB-453 cells. Mean ± SD of three independent experiments. *** *p* < 0.001, two-tailed Student’s *t*-test. (**E**) Allele-specific enrichment of rs4808611 site in FAIRE DNA determined by AS-qPCR in MDA-MB-468 cells. Mean ± SD of three independent experiments. *** *p* < 0.001, two-tailed Student’s *t*-test. (**F**) Chromatin structure and feature visualization for a 54kb rs4808611-centered region, including ChIP-seq signal intensity tracks of H3K27ac ChIP-seq experiments in MCF7 (green), MDA-MB-453 (red), MDA-MB-468 (blue) and ZR-75-1 (dark blue) cells and H3K4me3 ChIP-seq experiment in ZR-75-1 (dark blue) cells. Chromatin structure (top) and gene information (bottom) are shown, with the SNP position marked by a vertical line. (**G**) ChIP-qPCR analysis of the rs4808611 region in H3K27ac ChIP DNA in MCF7 cells. Mean ± SD of three technical replicates. *** *p* < 0.001, two-tailed Student’s *t*-test. (**H**) NGS read counts of the rs4808611 alleles in H3K27ac ChIP-seq analysis in the MDA-MB-453 cell line. (**I**) NGS read counts of the rs4808611 alleles in H3K27ac ChIP-seq analysis in the MDA-MB-468 cell line.

We then systematically investigated the roles and molecular mechanisms herein in breast cancer susceptibility for given sites. We first confirmed the allele-specific gene regulatory activity displayed in DiR-seq analysis (Figure 3F and Figure S4A–D) with DiR-qPCR analysis (Figure 5A and Figure S4E–H). Then, we cloned the 733bp genomic region surrounding the rs4808611 site and performed a reporter assay in MCF7 cells. Consistently, the C allele exhibited significantly higher activity than the T allele (Figure 5B) in MCF7 cells. Furthermore, we investigated the chromatin open status of the rs4808611 region in ten breast cancer cell lines and found dramatic enrichment of this region in FAIRE DNA in all ten cells (Figure 5C). Interestingly, AS-qPCR analysis of the FAIRE DNA in the heterozygous cells, MDA-MB-453 and MDA-MB-468, showed that the C allele was significantly preferred in open chromatin compared to the T allele (Figure 5D, E). Moreover, our ChIP-seq analysis indicated that the rs4808611 site was enriched in the H3K27ac modification in four breast cancer cell lines, including MCF7, MDA-MB-453, MDA-MB-468, and ZR-75-1 (Figure 5F). We also performed the qPCR analysis with ChIP DNA and found that the rs4808611 region was highly enriched in the H3K27ac histone modification in both MDA-MB-453 and MDA-MB-468 cells (Figure 5G). Notably, the C allele was also preferred in the H3K27ac ChIP DNA in both MDA-MB-453 (Figure 5H) and MDA-MB-468 cells (Figure 5I) as determined by NGS read count. These results indicate that rs4808611 is supposed to possess important gene regulatory activity in breast cancer cells.

### 3.5. rs4808611 Alters Gene Expression of NR2F6

**Figure 6:**
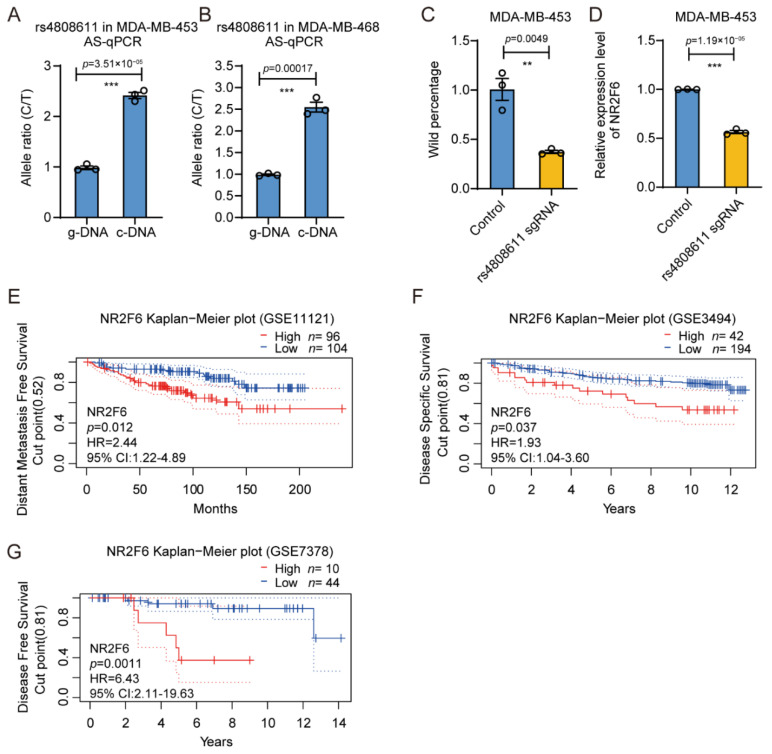
rs4808611 affects gene expression of *NR2F6*. (**A**,**B**) Allele preference in the transcription of the rs4808611 region in the MDA-MB-453 (**A**) and MDA-MB-468 (**B**) cells was determined by AS-qPCR. Mean ± SD of three technical replicates. *** *p* < 0.001, two-tailed Student’s *t*-test. (**C**) The frequency of CRISPR/Cas9 genome editing on the rs4808611 region, determined by getPCR analysis in MDA-MB-453 cells. Mean ± SD of three technical replicates. ** *p* < 0.01, two-tailed Student’s *t*-test. (**D**) Gene expression level of *NR2F6* in the genome-edited MDA-MB-453 cells determined by RT-qPCR and normalized with *ACTB* expression. Mean ± SD of three technical replicates. *** *p* < 0.001, two-tailed Student’s *t*-test. (**E**) Kaplan–Meier plot showing the distant metastasis-free survival of the breast cancer patients from the GEO cohort (GSE11121), stratified according to the expression level of gene *NR2F6* (Cut point = 0.52). (**F**) Kaplan–Meier plot showing the disease-specific survival of breast cancer patients from the GEO cohort (GSE3494), stratified by the expression level of gene *NR2F6* with a cut point of 0.81. (**G**) Kaplan–Meier plot showing the disease-free survival of breast cancer patients in the GEO cohort (GSE7378), grouped by the expression level of gene *NR2F6* with a cut point of 0.81. The HR was provided with a 95% confidence interval, and *p* values were calculated with the log-rank test for (**E**–**G**).

To confirm the regulatory role of the rs4808611 site on the *NR2F6* gene, we performed AS-qPCR analysis with cDNA and genomic DNA as templates. Even though located in the intron, we still observed a strong allele preference for the C allele when the rs4808611 was transcribed in MDA-MB-453 (Figure 6A) and MDA-MB-468 (Figure 6B) cell lines. In order to further investigate the direct regulatory relationship between rs4808611 and *NR2F6*, we destroyed the SNP sequence by using CRISPR/Cas9 technology in MDA-MB-453 cells (Figure 6C) and found that the expression of *NR2F6* significantly decreased upon editing (Figure 6D). Moreover, the Kaplan–Meier survival analysis demonstrated that patients with higher expression levels of *NR2F6* exhibited worse distant metastasis-free survival probability (Figure 6E), worse disease-specific survival probability (Figure 6F), and worse disease-free survival probability (Figure 6G). In brief, the results indicate that the breast cancer risk SNP rs4808611 promotes the gene expression of *NR2F6* and then results in a poor prognosis for breast cancer patients.

### 3.6. The Gene Regulatory Activity of rs2236007

**Figure 7:**
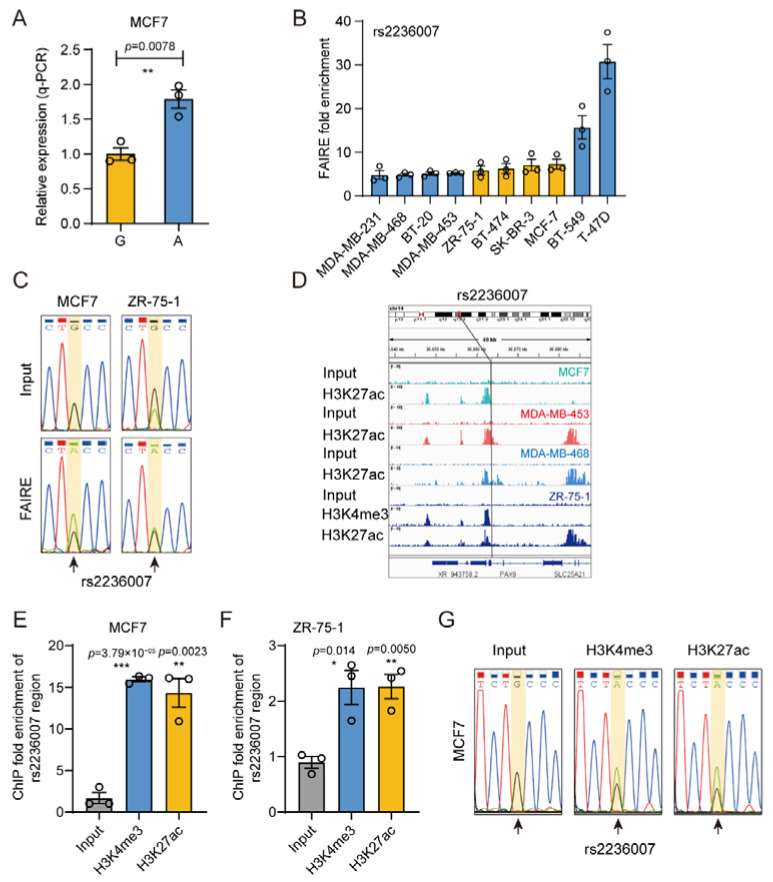
The gene regulatory activity analysis of rs2236007. (**A**) Allele-specific reporter gene expression level for rs2236007 SNP region in the DiR-qPCR assay in MCF7 cells. Mean ± SD of three independent experiments. ** *p* < 0.01, two-tailed Student’s *t*-test. (**B**) FAIRE-qPCR analysis of rs2236007 in ten breast cancer cells, including MDA-MB-231, MDA-MB-468, BT-20, MDA-MB-453, ZR-75-1, BT-474, SK-BR-3, MCF7, BT-549 and T-47D. Four cell lines heterozygous for rs2236007 are highlighted in orange. Mean ± SD of three independent experiments. (**C**) Sanger sequencing chromatography of the rs2236007 site region for input DNA and FAIRE DNA in MCF7 and ZR-75-1 cells. The position of rs2236007 is indicated with a yellow square background. Representative images from triplicate experiments. (**D**) Chromatin structure and feature visualization for a 49kb rs2236007-centered region, including ChIP-seq signal intensity tracks of H3K27ac ChIP-seq experiments in MCF7 (green), MDA-MB-453 (red), MDA-MB-468 (blue) and ZR-75-1 (dark blue) cells and H3K4me3 ChIP-seq experiment in ZR-75-1 (dark blue) cells. Chromatin structure (top) and gene information (bottom) are shown, with the SNP position marked by a vertical line. (**E**,**F**) ChIP-qPCR enrichment analysis of the rs2236007 region in H3K4me3 and H3K27ac ChIP DNA in MCF7 (**E**) and ZR-75-1 (**F**) cells. Mean ± SD of three technical replicates. *** *p* < 0.001, ** *p* <0.01, * *p* < 0.05, two-tailed Student’s *t*-test. (**G**) Sanger sequencing chromatography of the rs2236007 region for H3K27ac and H3K4me3 ChIP DNA and input DNA in MCF7 cells. The position of rs2236007 is indicated with a yellow square background.

When it turns to the functional SNP rs2236007, we also performed DiR-qPCR (Figure 7A) report assays in MCF7 cells and found that consistently with the DiR-seq results, the A allele exhibited significantly higher activity than the G allele. In addition, the rs2236007 site also showed comparable reporter activity in DiR-seq analysis of the MDA-MB-453 (Figure S5A), BT-474 (Figure S5B), and T-47D (Figure S5C) cell lines. In the FAIRE qPCR analysis for determining the open status of the rs2236007 region, we found that this region was highly enriched in the FAIRE DNA in ten different breast cancer cells (Figure 7B). Interestingly, Sanger sequencing chromatography of the FAIRE DNA showed strong allele preference in the heterozygous cell lines MCF7 and ZR-75-1 (Figure 7C) but not in BT-474 (Figure S5D) or SK-BR-3 (Figure S5E). Moreover, our ChIP-seq analysis using anti-H3K27ac and anti-H3K4me3 antibodies in multiple cell lines showed significant enrichment of the rs2236007 region (Figure 7D). Further qPCR analysis of the ChIP DNA confirmed the enrichment of this site in H3K27ac modification in MCF7 (Figure 7E) and ZR-75-1 (Figure 7F) cells. The Sanger sequencing chromatography of the H3K27ac ChIP DNA showed a strong allele preference for the A allele in MCF7 cells (Figure 7G), which was not observed in the ZR-75-1 (Figure S5F) cell line. The possible reason may be that the ChIP fold enrichment of the site rs2236007 was far lower in ZR-75-1 than MCF7 cells. All these results demonstrate that rs2236007 plays an essential regulatory role in breast cancer cells.

### 3.7. rs2236007 Affects PAX9 Expression by Altering EGR1 Binding

**Figure 8:**
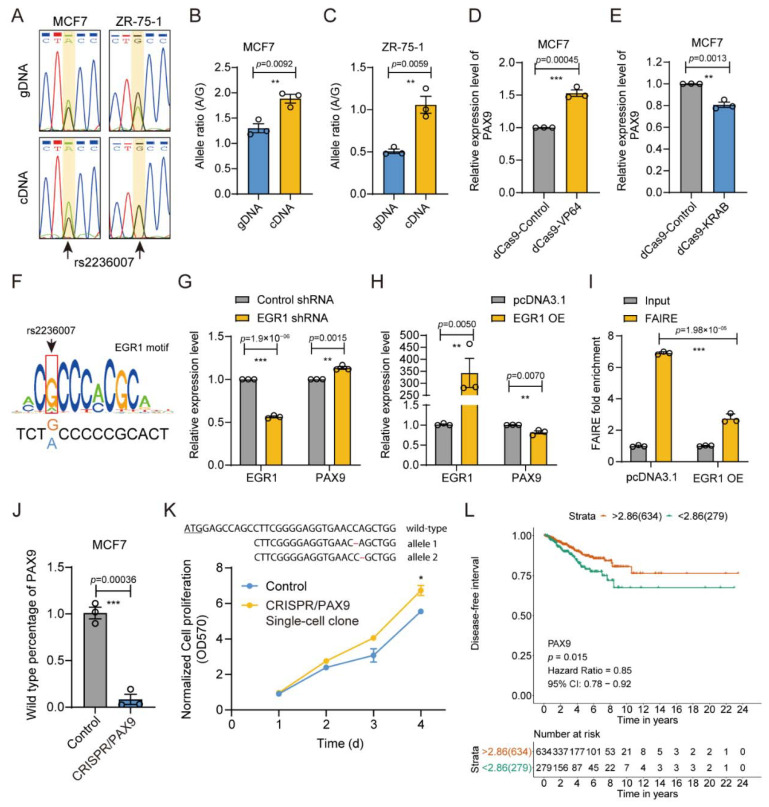
The rs2236007 variation alters *PAX9* expression. (**A**) Sanger sequencing chromatography of the rs2236007 region for genomic DNA and cDNA of MCF7 and ZR-75-1 cells. The position of rs2236007 is highlighted in a yellow square. Representative images from triplicate experiments. (**B**,**C**) Allele preference analysis of rs2236007 site in genomic DNA and cDNA determined by AS-qPCR in MCF7 (**B**) and ZR-75-1 (**C**) cells. Mean ± SD of three technical replicates. ** *p* < 0.01, two-tailed Student’s *t*-test. (**D**) CRISPR-mediated activation of the *PAX9* gene using dCas9-VP64 guided to the rs2236007 region in MCF7 cells determined by RT-qPCR. Mean ± SD of three independent experiments. *** *p* < 0.001, two-tailed Student’s *t*-test. (**E**) CRISPR interference of the *PAX9* gene using dCas9-KRAB guided to the rs2236007 region in MCF7 cells determined by RT-qPCR. Mean ± SD of three independent experiments. ** *p* < 0.01, two-tailed Student’s *t*-test. (**F**) PWM motif prediction in the JASPAR database showing the G allele-preferred binding of EGR1 to the rs2236007 region. (**G**) The effect of *ERG1* shRNA knockdown on the gene expression of *PAX9* in MCF7 cells was determined by RT-qPCR. Mean ± SD of three independent experiments. ** *p* < 0.01, *** *p* < 0.001, two-tailed Student’s *t*-test. (**H**) The effect of EGR1 overexpressed on *PAX9* expression in MCF7 cells was determined by RT-qPCR. Mean ± SD of three technical replicates. ** *p* < 0.01, two-tailed Student’s *t*-test. (**I**) FAIRE-qPCR analysis showing the impact of EGR1 overexpression on the openness of the rs2236007 region in MCF7 cells. Mean ± SD of three technical replicates. *** *p* < 0.001, two-tailed Student’s *t*-test. (**J**) Evaluation of genome editing frequency on the *PAX9* gene in MCF7 cells determined with getPCR analysis. Mean ± SD of three technical replicates. *** *p* < 0.001, two-tailed Student’s *t*-test. (**K**) Cell proliferation assay of MCF7 single-cell clone with *PAX9* knocked out through CRISPR/Cas9. OD570 was acquired using the PrestoBlue^®®^ Cell Viability Reagent. The genotype of the clone (top) indicating one-base deletion variations on both alleles. Start codon ATG is highlighted with an underline. Mean ± SD of three biological replicates. * *p* < 0.05, two-tailed Student’s *t*-test. (**L**) Kaplan–Meier survival analysis showing the disease-free interval of breast cancer patients from the TCGA cohort, stratified according to the expression level of *PAX9* (strata point = 2.68). The HR was provided with a 95% confidence interval, and *p* values were calculated with the log-rank test.

In order to verify the regulatory relationship between rs2236007 and the related gene *PAX9*, we evaluated the allele ratio of rs2236007 in MCF7 cDNA compared to genomic DNA by using Sanger sequencing chromatography (Figure 8A) and AS-qPCR analysis (Figure 8B,C). We found that the A allele was significantly preferred in the transcribed RNA product in MCF7 and ZR-75-1 cell lines. In the online eQTL analysis with the Genotype-Tissue Expression (GTEx) database, we found that the *PAX9* gene was highly associated with the rs2236007 variation in the thyroid, tibial, and skin tissues (Figure S6), with the A/A genotype correlated with the high expression of *PAX9*. In order to further confirm the regulation role of rs2236007 on the *PAX9* gene, we performed chromatin modulation of the rs2236007 region with the dCas9-VP64 or dCas9-KRAB tools [47]. The dCas9-VP64 treatment significantly increased the gene expression level of *PAX9* (Figure 8D), and dCas9-KRAB decreased *PAX9* expression relative to the contrary (Figure 8E).

Then, we explored the potential transcription factors that participated in the biological function of the rs2236007 site by scanning the rs2236007 genomic sequence with position weight matrix (PWM) of all transcription factors in the JASPAR database [72]. We found that EGR1 was the potential transcription factor that bound the rs2236607 region, and the G allele promoted EGR1 binding (Figure 8F). Notably, EGR1 knockdown with shRNA significantly resulted in upregulation of the *PAX9* gene (Figure 8G), and the overexpression of EGR1 caused obvious downregulation of the *PAX9* gene in MCF7 cells (Figure 8H). Correspondingly, the overexpression of EGR1 significantly decreased the enrichment of the rs2236007 region in FAIRE DNA in MCF7 cells (Figure 8I). All these results indicate that the G allele of the rs2236007 site could downregulate the gene expression of *PAX9* by promoting the binding of the suppressive transcription factor EGR1.

In order to further investigate the biological function of the target gene *PAX9*, we assessed the effect of the *PAX9* gene on cancerous phenotypes in MCF7 cells. We edited the coding region of the gene *PAX9* using the CRISPR/Cas9 technology in MCF7 cells by an indel frequency of up to 95% (Figure 8J). Then, we isolated single-cell clones and obtained a clone with one C deleted at 28bp downstream of the start codon and one A base deleted on the other allele at 29bp downstream of the transcription start codon (Figure 8K). The single-base deletion could result in frameshift mutation of the *PAX9* gene on both alleles. We found that the destruction of the *PAX9* gene increased cell proliferation significantly (Figure 8K). Notably, the Kaplan–Meier survival analysis demonstrated that patients with lower expression levels of *PAX9* exhibited worse relapse-free survival probability (Figure 4F) and disease-free survival probability (Figure 8L).

In brief, the results indicate that the rs2236007 downregulates the gene expression of *PAX9* by affecting the binding of suppressive transcription factor EGR1 and contributes to the malignancy of breast cancer with a poor prognosis for breast cancer patients.

## 4. Discussion

This study applied DiR-seq analysis in nine breast cancer cell lines to screen causal risk SNPs that possess potential gene regulatory functions. The functional SNPs picked out from the nine breast cancer cells are strongly cell-specific. In order to further nominate the most plausible SNPs, we integrated the DiR-seq results with the multi-omics analysis characterizing chromatin open and active status and finally defined seven functional variants from 288 breast cancer GWAS SNPs. All seven variants altered transcription regulatory activity in an allele-specific manner and are enriched in the active chromatin regions in MCF7 cells. Notably, the related genes of the seven SNPs have significant associations with the relapse-free survival probability for breast cancer patients. Our integrative analysis of the multi-omics data lays solid ground for thoroughly understanding the role and mechanism of the breast cancer risk SNPs.

Further systematic investigation shed light on the roles and underlying mechanisms for rs4808611 and rs2236007 in breast cancer susceptibility. For the functional site rs4808611, the risk allele C upregulates the *NR2F6* gene and contributes to a poor prognosis for breast cancer patients. For the rs2236007 site, the risk allele G downregulates the *PAX9* gene via recruiting the suppressive transcription factor EGR1 and results in malignancy and poor prognosis for breast cancer patients.

In our analysis on gene *NR2F6*, we found that the higher expression of *NR2F6* was significantly associated with the lower survival probability for breast cancer patients. Similar to our inquiry in breast cancer, *NR2F6* has already been proven upregulated frequently in bladder cancer tissues compared with their paired normal tissues [73]. It has been reported that *EGR1* played an essential role in the development of tumor diseases. Compared to normal tissues, the expression of *EGR1* decreased in colon cancer, ovarian cancer, and liver cancer tissues, and the ectopic expression of the *EGR1* gene could reduce the migration of cancer cells [74,75,76]. Targeting the *EGR1* with DNAzymes significantly inhibited the growth of breast cancer solid tumors [77]. This indicates that the *EGR1* gene should play an essential role in breast cancer progression. We proved that the lower expression of *PAX9* could promote the cell proliferation of breast cancer cells. Moreover, the lower expression of *PAX9* is significantly associated with the lower survival probability for breast cancer patients. This indicates that the *PAX9* gene should be an essential marker candidate in breast cancer management.

However, more efforts might be needed to understand the function of the rs4808611 and rs2236007 sites fully. For the rs4808611 site, future works might include deciphering the binding transcription factors that mediate the regulatory effect on the *NR2F6* gene and investigating the effect of the *NR2F6* gene on cancerous phenotypes. As to the rs2236007 site, the mechanism of *PAX9* in terms of resulting in malignancy and poor prognosis of breast cancer is of great importance before the understanding can enter clinical transformation in breast cancer.

## 5. Conclusions

In general, we nominated seven potential regulatory SNPs associated with breast cancer susceptibility by integrative analysis of DiR-seq reporter assays and chromatin profiling omics analysis in breast cancer cell lines. Moreover, we elucidated the roles and molecular mechanism of rs4808611 and rs2236007 herein in terms of resulting in breast cancer risk. The results described here could be valuable for understanding the roles of the GWAS SNPs for breast cancer patients.

## Data Availability

The raw sequence data and processed data generated using the Illumina Hiseq-PE150 platform for DiR-seq assay in nine breast cancer cell lines have been made publicly available in the Gene Expression Omnibus (GEO) database under the accession number GSE178198.

## Supplementary Materials

The following are available online at https://www.mdpi.com/article/10.3390/cimb43030124/s1, Figure S1: DiR-seq analysis of breast cancer risk SNPs in different breast cancer cells, Figure S2: Volcano plots of DiR-seq results for the seven breast cancer cells, Figure S3: Multi-omics analysis nominated the most functional variants, Figure S4: Reporter gene expression level of rs4808611 site in breast cancer cells, Figure S5: The allelic activity analysis of rs2236007, Figure S6: The eQTL analysis of different tissues in GTEx reveals the association between alleles of rs2236007 and *PAX9* gene, Table S1: 285 GWAS SNPs information, Table S2: Three SNPs information from a review paper, Table S3: The SNP-centered DNA sequence, Table S4: 288 SNPs with their locations, Table S5: The primers for DiR-seq library preparation, Table S6: The functional SNPs in nine breast cancer cells, Table S7: The tested primers of DiR-seq system, Table S8: qPCR primers and genotyping primers, Table S9: The primers for CRISPR assay and clones construction, Table S10: Multi-omics analysis results in T-47D, Table S11: Multi-omics analysis results in MCF7.
