# Supplementary material for: Functional Screenings Identify Regulatory Variants Associated with Breast Cancer Susceptibility
Source: Curr Issues Mol Biol. 2021 Oct 26;43(3):1756–77. doi: 10.3390/cimb43030124 (PMC8928974; doi:10.3390/cimb43030124)
Supplement: Supplementary file 1 [file cimb-43-00124-s001.zip › Supplementary Figures.pdf]

Figure S1. DiR-seq analysis of breast cancer risk SNPs in different breast cancer cells.

Figure S2. Volcano plots of DiR-seq results for the seven breast cancer cells.

Figure S3. Multi-omics analysis nominated the most functional variants.

Figure S4. Reporter gene expression level of rs4808611 site in breast cancer cells.

Figure S5. The allelic activity analysis of rs2236007.

Figure S6. The eQTL analysis of different tissues in GTEx reveals the association between alleles of rs2236007 and the *PAX9* gene.

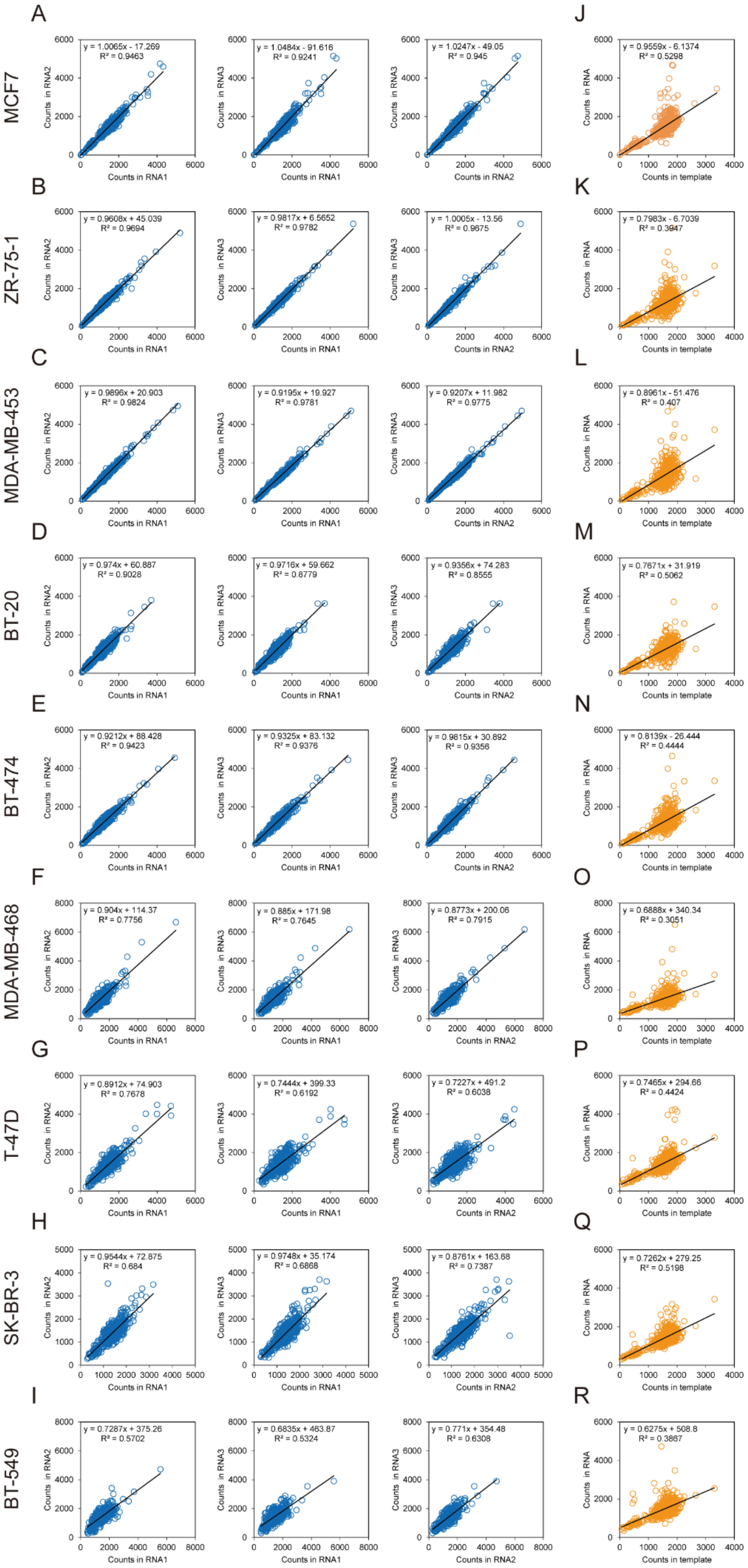
A
B
C
D
E
F
G
H
I
J
K
L
M
N
O
P
Q
R
MCF7
ZR-75-1
MDA-MB-453
BT-20
BT-474
MDA-MB-468
T-47D
SK-BR-3
BT-549
Counts in RNA1
Counts in RNA2
Counts in RNA3
Counts in RNA
Counts in template
y = 1.0065x - 17.269
R² = 0.9463
y = 1.0484x - 91.616
R² = 0.9241
y = 1.0247x - 49.05
R² = 0.945
y = 0.9559x - 6.1374
R² = 0.5298
y = 0.9608x + 45.039
R² = 0.9694
y = 0.9817x + 6.5652
R² = 0.9782
y = 1.0005x - 13.56
R² = 0.9675
y = 0.7983x - 6.7039
R² = 0.3947
y = 0.9896x + 20.903
R² = 0.9824
y = 0.9195x + 19.927
R² = 0.9781
y = 0.9207x + 11.982
R² = 0.9775
y = 0.8961x - 51.476
R² = 0.407
y = 0.974x + 60.887
R² = 0.9028
y = 0.9716x + 59.662
R² = 0.8779
y = 0.9356x + 74.283
R² = 0.8555
y = 0.7671x + 31.919
R² = 0.5062
y = 0.9212x + 88.428
R² = 0.9423
y = 0.9325x + 83.132
R² = 0.9376
y = 0.9815x + 30.892
R² = 0.9356
y = 0.8139x - 26.444
R² = 0.4444
y = 0.904x + 114.37
R² = 0.7756
y = 0.885x + 171.98
R² = 0.7645
y = 0.8773x + 200.06
R² = 0.7915
y = 0.6888x + 340.34
R² = 0.3051
y = 0.8912x + 74.903
R² = 0.7678
y = 0.7444x + 399.33
R² = 0.6192
y = 0.7227x + 491.2
R² = 0.6038
y = 0.7465x + 294.66
R² = 0.4424
y = 0.9544x + 72.875
R² = 0.684
y = 0.9748x + 35.174
R² = 0.6868
y = 0.8761x + 163.68
R² = 0.7387
y = 0.7262x + 279.25
R² = 0.5198
y = 0.7287x + 375.26
R² = 0.5702
y = 0.6835x + 463.87
R² = 0.5324
y = 0.771x + 354.48
R² = 0.6308
y = 0.6275x + 508.8
R² = 0.3867

Figure S1. DiR-seq analysis of breast cancer risk SNPs in different breast cancer cells. Correlation analysis between transfection replicates of DiR-seq assay for nine breast cancer cell lines, including MCF7, ZR-75-1, MDA-MB-453, BT-20, BT-474, MDA-MB-468, T-47D, SK-BR-3, and BT-549 (**A-I**). Scatter plot of DiR-seq tag counts in RNA and template in nine cell lines (**J-R**). The value for each tag is plotted with the tag count normalized to 1M and shown as a color circle. Trend lines of regression analysis and Pearson correlation coefficients are shown in the upper left corner.

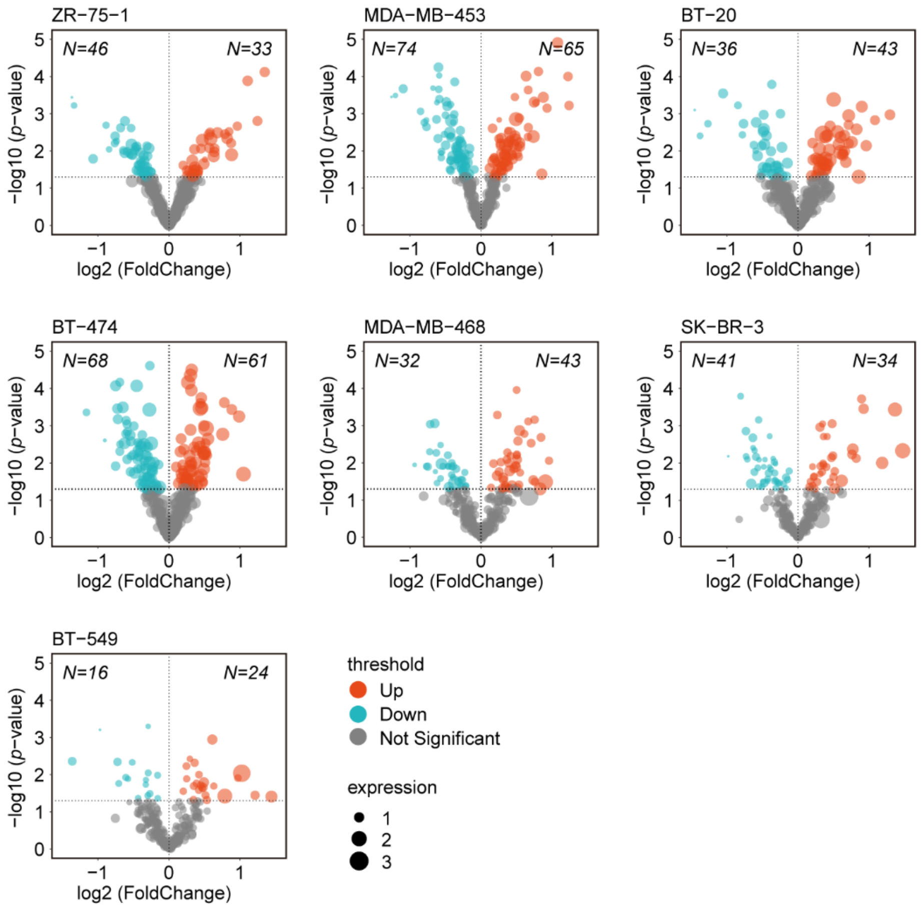

Figure S2. Volcano plots of DiR-seq results for the seven breast cancer cells. *p* values came from a two-tailed Student's t-test of effect sizes of two alleles. Dashed horizontal lines indicate the p-value < 0.05 cutoff. The regulatory SNPs exhibiting increased activity with the risk allele are shown in orange; those with decreased activity are shown in blue. Gray dots represent SNPs showing no significant difference between alleles.

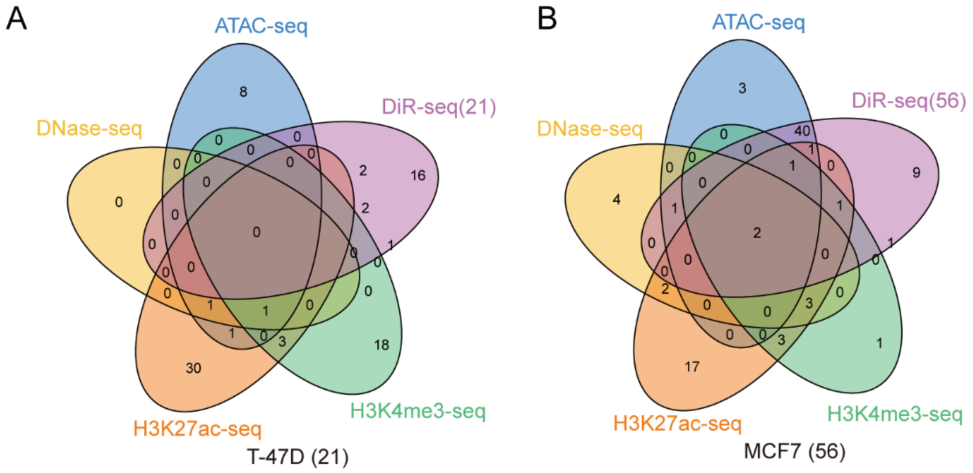

Figure S3. Multi-omics analysis nominated the most functional variants.

(A) Venn diagram illustrated the putative functional SNPs in DiR-seq analysis of T-47D cells and relevant omics signatures, including ATAC-seq, Dnase-seq, H3K27ac-seq, and H3K4me3 obtained from ENCODE database or GEO database. The accession number is seen in Figure 2A legend. **(B)** Venn diagram illustrated the putative functional SNPs in DiR-seq analysis of MCF7 cells and relevant omics signatures, including ATAC-seq, Dnase-seq, H3K27ac-seq, and H3K4me3 obtained from ENCODE database. The accession number is seen in Figure 2B legend.

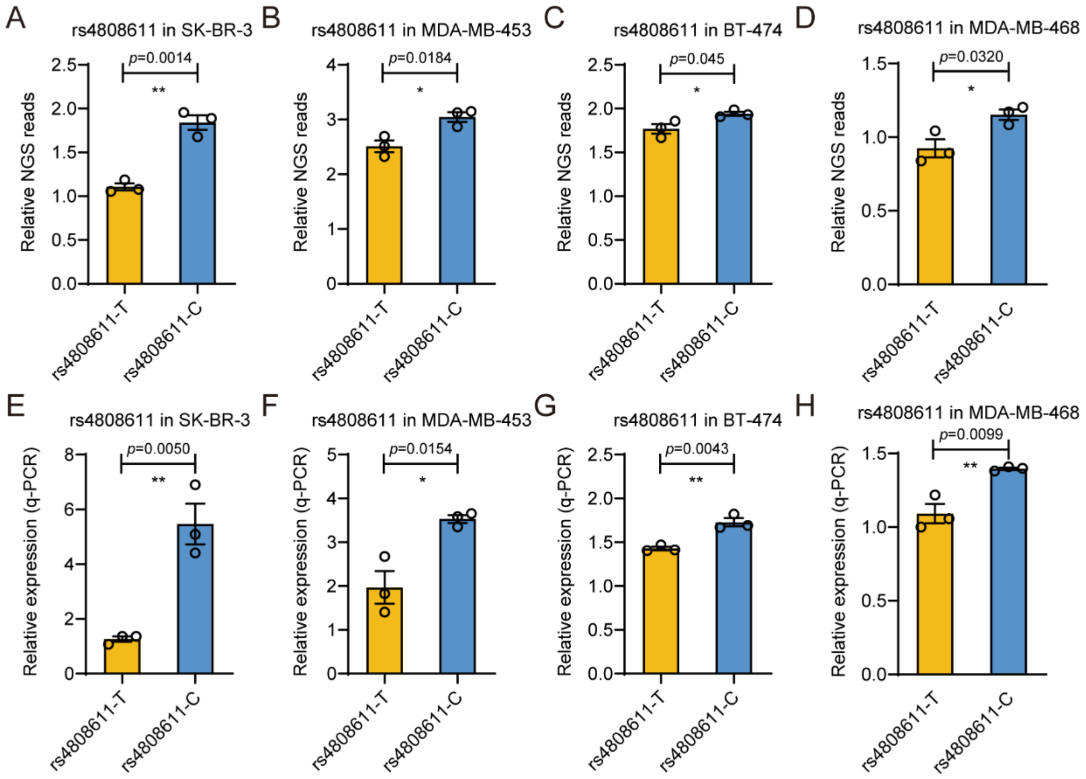

Figure S4. Reporter gene expression level of rs4808611 site in breast cancer cells.
Reporter gene expression level for rs4808611 SNP region in the DiR-seq analysis in SK-BR-3**(A)**, MDA-MB-453**(B),** BT-474**(C),** and MDA-MB-468**(D)** cells. C allele showed increased enhancer activity relative to the T allele. Mean ± SD of three independent experiments. *$p<0.05$, **$p<0.01$, two-tailed Student's *t*-test. Reporter gene expression level for rs4808611 SNP region in the DiR-qPCR assay in SK-BR-3**(E)**, MDA-MB-453**(F)**, BT-474**(G),** and MDA-MB-468**(H)** cells. Mean ± SD of three independent experiments. *$p<0.05$, **$p<0.01$, two-tailed Student's *t*-test.

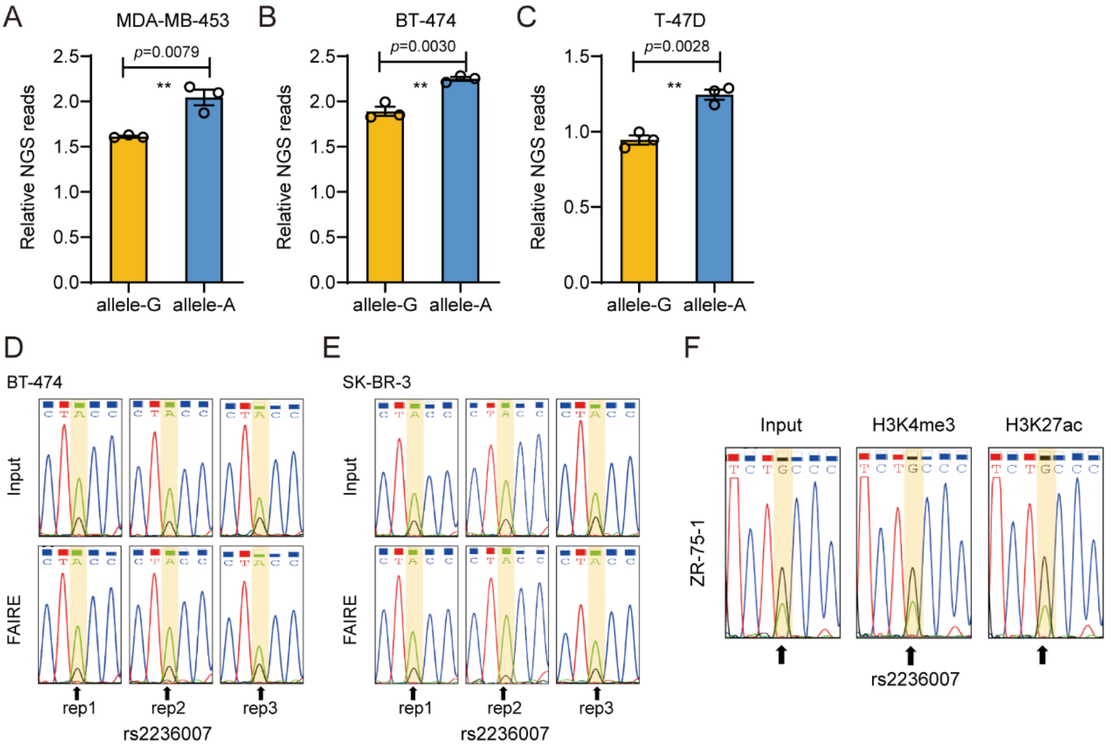

Figure S5. The allelic activity analysis of rs2236007.

(A) Reporter gene expression level for rs2236007 SNP region in the DiR-seq analysis in MDA-MB-453 cells. G allele showed decreased enhancer activity relative to the A allele. Mean ± SD of three independent experiments. **p<0.01, two-tailed Student's t-test. **(B)** Reporter gene expression level for rs2236007 SNP region in the DiR-seq analysis in BT-474 cells. G allele showed decreased enhancer activity relative to the A allele. Mean ± SD of three independent experiments. **p<0.01, two-tailed Student's t-test. **(C)** Reporter gene expression level for rs2236007 SNP region in the DiR-seq analysis in T-47D cells. G allele showed decreased enhancer activity relative to the A allele. Mean ± SD of three independent experiments. **p<0.01, two-tailed Student's t-test. **(D)** Sanger sequencing chromatography allelic analysis of Input DNA and FAIRE DNA for the rs2236007 site in BT-474. The position of rs2236007 is highlighted in a yellow square. **(E)** Sanger sequencing chromatography allelic analysis of Input DNA and FAIRE DNA for the rs2236007 site in SK-BR-3. The position of rs2236007 is highlighted in a yellow square. **(F)** Sanger sequencing chromatography allelic analysis of rs2236007 in H3K27ac and H3K4me3 ChIP DNA and Input DNA in ZR-75-1 cells. The position of rs2236007 is highlighted in a yellow square.

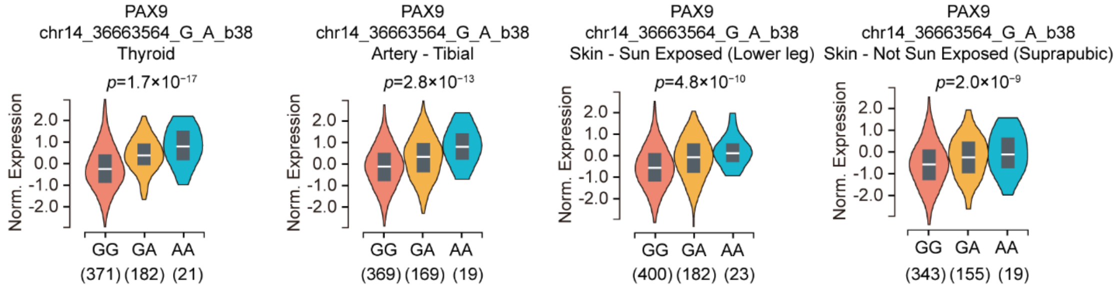

Figure S6. The eQTL analysis of different tissues in GTEx reveals the association between alleles of rs2236007 and the *PAX9* gene. *P* values are from a linear regression model.
